# Chloroplastic thioredoxin-*f* and thioredoxin-*m*1/4 play important roles in brassinosteroids-induced changes in CO_2_ assimilation and cellular redox homeostasis in tomato

**DOI:** 10.1093/jxb/eru207

**Published:** 2014-05-20

**Authors:** Fei Cheng, Yan-Hong Zhou, Xiao-Jian Xia, Kai Shi, Jie Zhou, Jing-Quan Yu

**Affiliations:** ^1^Department of Horticulture, Zijingang Campus, Zhejiang University, Yuhangtang Road 866, Hangzhou, 310058, PR China; ^2^Key Laboratory of Horticultural Plants Growth, Development and Quality Improvement, Agricultural Ministry of China, Yuhangtang Road 866, Hangzhou, 310058, PR China

**Keywords:** Antioxidant, Benson–Calvin cycle, chloroplast, 2-Cys peroxiredoxin, glutathione, photosynthesis.

## Abstract

Chloroplast thioredoxins (TRXs) and glutathione function as redox messengers in the regulation of photosynthesis. In this work, the roles of chloroplast TRXs in brassinosteroids (BRs)-induced changes in cellular redox homeostasis and CO_2_ assimilation were studied in the leaves of tomato plants. BRs-deficient *d* ^*^im*^ plants showed decreased transcripts of *TRX-f*, *TRX-m2*, *TRX-m1/4*, and *TRX-x*, while exogenous BRs significantly induced CO_2_ assimilation and the expression of *TRX-f*, *TRX-m2*, *TRX-m1/4*, and *TRX-x*. Virus-induced gene silencing (VIGS) of the chloroplast *TRX-f*, *TRX-m2*, *TRX-m1/4*, and *TRX-y* genes individually increased membrane lipid peroxidation and accumulation of 2-Cys peroxiredoxin dimers, and decreased the activities of the ascorbate–glutathione cycle enzymes and the ratio of reduced glutathione to oxidized glutathione (GSH/GSSG) in the leaves. Furthermore, partial silencing of *TRX-f*, *TRX-m2*, *TRX-m1/4*, and *TRX-y* resulted in decreased expression of genes involved in the Benson–Calvin cycle and decreased activity of the associated enzymes. Importantly, the BRs-induced increase in CO_2_ assimilation and the increased expression and activities of antioxidant- and photosynthesis-related genes and enzymes were compromised in the partially *TRX-f*- and *TRX-m1/4*-silenced plants. All of these results suggest that TRX-*f* and TRX-*m*1/4 are involved in the BRs-induced changes in CO_2_ assimilation and cellular redox homeostasis in tomato.

## Summary

Virus-induced gene silencing (VIGS) was used in this study to characterize the role of thioredoxin-*f* and thioredoxin-*m*1/4 in brassinosteroid-induced changes in CO_2_ assimilation and cellular redox homeostasis in tomato.

## Introduction

Accumulating evidence supports the hypothesis that the cellular redox signalling and hormone signalling pathways form an integrated redox–hormone network that regulates many plant growth- and defence-related pathways (Bartoli *et al.*, 2013). Redox regulation is often mediated by thioredoxins (TRXs), which are able to (de-)activate enzymes through the reversible reduction of disulphide bonds (Buchanan and Balmer, 2005; Montrichard *et al.*, 2009).

TRXs are ubiquitous, low molecular weight (~12kDa) proteins that catalyse thiol–disulphide exchange reactions in conjunction with a large number of enzymes and related proteins (Jacquot *et al.*, 2002). The short peptide motif WC(G/P)PC, with two conserved cysteine residues, has been characterized as the conserved active redox site of TRXs (Jacquot *et al.*, 1997; Buchanan and Balmer, 2005; Meyer *et al.*, 2008). Plant cells contain numerous TRXs in the cytosol, nucleus, mitochondria, endoplasmic reticulum, and chloroplasts, which continue to be identified (Marcus *et al.*, 1991; Baumann and Juttner, 2002; Meyer *et al.*, 2005). Recent proteomic studies using TRX-trapping chromatography or labelled gel electrophoresis in combination with protein identification via mass spectrometry have identified >180 potential *in vitro* TRX target proteins (Motohashi *et al.*, 2001; Balmer *et al*., 2003, 2004, 2006; Wong *et al.*, 2004; Marchand *et al.*, 2006; Alkhalfioui *et al.*, 2007).

There are five types of typical TRXs present in the chloroplasts. The *f*- and *m*-type TRXs function as messengers in the Fdx (ferredoxin)/TRX system by transmitting the redox signal from Fdx:TRX reductase to the target enzymes. TRX-*f* has been shown to activate several chloroplast enzymes, including enzymes involved in the Benson–Calvin cycle, ATP synthesis, and fatty acid synthesis (Nishizawa and Buchanan, 1981; Wolosiuk *et al.*, 1993; Sasaki *et al.*, 1997; Schwarz *et al.*, 1997; Zhang and Portis, 1999). TRX-*m* has been found to be involved in the inactivation of glucose-6-phosphate dehydrogenase, which is the first enzyme in the oxidative pentose phosphate cycle (Wenderoth *et al.*, 1997). Both TRX-*f* and TRX-*m* can activate fructose-1,6-bisphosphatase (FBPase) and NADP-dependent malate dehydrogenase (NADP-MDH) *in vitro*, suggesting that there is some overlap in substrate specificity between the isoforms (Hodges *et al.*, 1994; Geck *et al.*, 1996). TRX-*x*, which is inactive toward FBPase and NADP-MDH, is the most efficient reductant of 2-Cys peroxiredoxin (2-CP). This property suggests that TRX-*x* functions specifically in resistance to oxidative stress (Collin *et al.*, 2003). TRX-*y* exists in two isoforms and has been reported to act as an efficient electron donor for the chloroplast glutathione peroxidase (Navrot *et al.*, 2006) and methionine sulphoxide reductase B2 (Vieira Dos Santos *et al.*, 2007). Peroxiredoxin Q, which has been reported to function as an antioxidant (Rouhier *et al.*, 2004; Lamkemeyer *et al.*, 2006), was shown to be the best substrate of TRX-*y* (Collin *et al.*, 2004). Lastly, TRX-*z* regulates plastid-encoded RNA polymerase-dependent transcription (Arsova *et al.*, 2010). However, the biological functions of TRXs have been studied mostly in *Arabidopsis* through *in vitro* experiments, and detailed *in vivo* studies using different isoforms have not yet been conducted.

Brassinosteroids (BRs) are a group of plant steroid hormones that have been shown to play important roles in the growth, development, and stress response of plants (Clouse and Sasse, 1998; Müssig, 2005). For example, the tomato *d* ^*^im*^ mutant with the block in C-6 oxidation in BRs biosynthesis results in a dwarf phenotype (Bishop *et al.*, 1999). Previous studies showed that BRs can enhance stress tolerance against chilling, paraquat (PQ), and biotic stress, and this effect is largely dependent on BRs-induced hydrogen peroxide (H_2_O_2_) accumulation in the apoplast (Xia *et al.*, 2009*b*). Furthermore, BRs are involved in the regulation of CO_2_ assimilation in several plants. In cucumber, BRs induce transient increases in *RBOH1* (*respiratory burst oxidase homolog 1*) expression, NADPH oxidase activity, H_2_O_2_ levels in the apoplast, and nitric oxide (NO) production (Xia *et al.*, 2009*b*; Cui *et al.*, 2011). Additionally, BRs can induce the expression and activity of genes and enzymes involved in the Benson–Calvin cycle, and this effect is attributed to the apoplastic H_2_O_2_-induced activation of photosynthesis-related redox-sensitive enzymes (Jiang *et al.*, 2012*a*). Apoplastic H_2_O_2_ can induce an increase in the ratio of reduced glutathione (GSH) to oxidized glutathione (GSSG), resulting in increased stability of redox-sensitive enzymes (Jiang *et al.*, 2012*b*). Microarray analysis revealed that several *TRX* genes are targeted by BRs in *Arabidopsis* and rice (Müssig *et al.*, 2002; Goda *et al.*, 2004; Wu *et al.*, 2008); however, their potential roles in BRs-induced cellular redox homeostasis and CO_2_ assimilation remain unknown.

Tomato is an important horticultural crop distributed world-wide and has been widely used to study the stress response. Virus-induced gene silencing (VIGS) has been well established and widely used for the analysis of gene functions in Solanaceous species (Liu *et al*., 2002*a*, *b*; Li *et al.*, 2006). In this study, the roles of the different chloroplastic TRXs in the BRs-induced changes in cellular redox homeostasis and CO_2_ assimilation were examined. Accordingly, five chloroplast TRXs were partially silenced individually using a VIGS approach, and the changes in CO_2_ assimilation as well as the activities of Calvin cycle and ascorbate (AsA)–GSH cycle enzymes and cellular glutathione redox homeostasis were subsequently determined in the presence or absence of exogenous BRs.

## Materials and methods

### Plant materials and experimental design

#### Experiment I 

Tomato seeds from wild-type (*Solanum lycopersicum* L. cv. Condine Red, CR) and its partially BRs-deficient *d* ^*^im*^ mutant were obtained from the Tomato Genetics Resource Center (University of California, Davis, CA, USA). These seeds were germinated and grown in a mixture of peat and vermiculite (1:1, v:v) under a 16h light (200 μmol m^–2^ s^–1^; at 25 °C), 8h dark (at 20 °C) cycle. To determine the role of BRs in the regulation of CO_2_ assimilation and the effects of BRs on the transcript levels of the chloroplastic *TRX* genes, plants at the four-leaf stage were sprayed with water or 0.2 μM EBR (24-epibrassinolide; Sigma, Santa Clara, CA, USA), one of the most active and stable forms of BRs. Twenty-four hours later, CO_2_ assimilation was measured in the third leaf from the bottom. Additionally, leaf samples were harvested from the water- or EBR-treated CR and *d* ^*^im*^ plants, frozen immediately in liquid nitrogen, and stored at –80 °C prior to gene expression analyses.

#### Experiment II 

Tomato (CR) seeds were germinated and grown in a mixture of peat and vermiculite (1:1, v:v) under a 16h light (200 μmol m^–2^ s^–1^; at 25 °C), 8h dark (at 20 °C) cycle. To examine the roles of the chloroplast TRXs in the regulation of CO_2_ assimilation and to determine whether they are involved in the BRs-induced increase in CO_2_ assimilation, VIGS was performed when the cotyledonary leaves were fully expanded but the true leaves had not yet appeared. Partially gene-silenced plants at the six-leaf stage were treated with 0.2 μM EBR via foliar spraying on all of the leaves; 10ml of this solution was applied per plant, and distilled water was used as a control. After ~24h, CO_2_ gas exchange was measured, and leaf samples were harvested for biochemical and gene expression analyses.

### VIGS constructs and *Agrobacterium*-mediated virus infection

Five chloroplast TRX isoforms were obtained, namely *TRX-f*, *TRX-m2*, *TRX-m1/4*, *TRX-x*, and *TRX-y*, from the tomato genome database using the BLAST tool (http://mips.helmholtz-muenchen.de/plant/tomato/index.jsp) based on their similarity to the characterized chloroplast TRX isoforms in *Arabidopsis*. To generate the *Tobacco rattle virus* (pTRV) VIGS constructs pTRV2-*TRX-f*, pTRV2-*TRX-m2*, pTRV2-*TRX-m1/4*, pTRV2-*TRX-x*, and pTRV2*-TRX-y*, which were used for partial silencing of the *TRX-f*, *TRX-m2*, *TRX-m1/4*, *TRX-x*, and *TRX-y* genes, respectively, fragments of the corresponding genes with sizes of 210, 231, 312, 285, and 251bp were PCR amplified from tomato cDNA with primers containing *Xho*I (CTCGAG) and *Sac*I (GAGCTC) restriction sites. All of the primers used are listed in Supplementary Table S1 available at *JXB* online. The resulting plasmids were subsequently introduced into *Agrobacterium tumefaciens* strain GV3101.

For virus infiltration, a mixed culture of *A. tumefaciens* carrying the pTRV1:pTRV2-target gene in a 1:1 ratio was infiltrated into fully expanded cotyledonary leaves of tomato plants (Ekengren *et al.*, 2003). An *Agrobacterium* culture carrying the empty pTRV2 vector was also infiltrated into a set of plants, which were used as a control. The inoculated plants were maintained at 20–22 °C in a growth chamber with a 16h daylength. After ~4 weeks, quantitative real time–PCR (qRT–PCR) was performed to determine the gene silencing efficiency before the plants were used in assays (Supplementary Fig. S1 available at *JXB* online).

### Leaf gas exchange measurements

Gas exchange analysis was conducted on the third leaf for Experiment I and on the fifth leaf for Experiment II using an open gas exchange system (LI-6400; LI-COR, Lincoln, NE, USA). The light-saturated rate of CO_2_ assimilation (*A*_sat_) was measured under an ambient CO_2_ concentration of 380 μmol mol^–1^ at a saturating photosynthetic photon flux density (1000 μmol m^–2^ s^–1^), at a leaf temperature of 25±1.5 °C and a relative air humidity of 80–90%. An assimilation versus intercellular CO_2_ concentration (*A*/*C*_i_) curve was determined according to von Caemmerer and Farquhar (1981). Assimilation was first measured at the ambient CO_2_ concentration under which the plants had grown. The atmospheric CO_2_ concentration was decreased to 50 μmol mol^–1^ in a stepwise manner and then returned to the growth concentration to check that the original rate could be regained; the rate was then finally increased stepwise to 2000 μmol mol^–1^ to complete the response curve. The maximum ribulose-1,5-bisphosphate carboxylase/oxygenase (Rubisco) carboxylation rates (*V*_c,max_) and maximum ribulose-1,5-bisphosphate (RuBP) regeneration rates (*J*_max_) were estimated from the *A*/*C*_i_ curves using the method described by Ethier and Livingston (2004).

### Determination of lipid peroxidation

Lipid peroxidation was estimated by measuring malondialdehyde (MDA) equivalents, according to the method described by Hodges *et al.* (1999). Leaf samples (0.3g) were ground in 3ml of ice-cold 25mM HEPES buffer (pH 7.8) containing 0.2mM EDTA and 2% polyvinylpyrrolidone (PVP). The obtained homogenates were centrifuged at 4 °C for 20min at 12000 *g*, and the resulting supernatants were used for analysis of MDA equivalents. The samples were mixed with 10% trichloroacetic acid (TCA) containing 0.65% 2-thiobarbituric acid (TBA) and heated at 95 °C for 25min. The content of MDA equivalents was corrected for non-MDA compounds by subtracting the absorbance at 532nm of a TBA-less solution containing the plant extract.

### Non-reducing SDS–PAGE and western blot analysis of 2-Cys peroxiredoxin

Total proteins were isolated from leaf tissues in a protein extraction buffer (100mM HEPES, pH 7.5, 5mM EDTA, 5mM EGTA, 10mM Na_3_VO_4_, 10mM NaF, 50mM β-glycerophosphate, 1mM phenylmethylsulphonyl fluoride, 10% glycerol, and 7.5% polyvinylpolypyrrolidone) supplemented with 10mM *N*-ethylmaleimide (NEM) (thiol-blocking reagent). After centrifugation at 13 000 *g* for 20min, the supernatants were transferred to clean tubes, quickly frozen in liquid nitrogen, and stored at –80 °C. The protein concentration in the extracts was determined using the Bio-Rad protein assay kit (Bio-Rad, Hercules, CA, USA), and bovine serum albumin (BSA) was used as a standard. Reducing agents and boiling were omitted in this protocol, as it is important to maintain the remaining disulphide bonds (Muthuramalingam *et al.*, 2010). Total protein samples (15 μg) supplemented with 5× loading buffer [225mM TRIS-HCl, pH 6.8, 5 % (w/v) SDS, 50% glycerol, 0.05 % bromophenol blue] were separated via 12% SDS–PAGE, and the redox state of 2-CP was detected through western blot analysis with a polyclonal antibody against 2-CP (Beijing Protein Innovation, Beijing, China). After incubation with a horseradish peroxidase (HRP)-linked secondary antibody (Cell Signaling Technology, Boston, MA, USA), the complexes on the blot were visualized using an enhanced chemiluminescence kit (Perkin Elmer, Wellesley, MA, USA), according to the manufacturer’s instructions. Band intensity was quantified using Quantity One software.

### Determination of Rubisco, Rubisco activase (RCA), and FBPase activity

Rubisco activity was measured spectrophotometrically by coupling 3-phosphoglyceric acid formation with NADH oxidation at 25 °C according to the method described by Lilley and Walker (1974), with some modifications. Total activity was assayed after the crude extract had been activated in a 0.1ml activation mixture containing 33mM TRIS-HCl (pH 7.5), 0.67mM EDTA, 33mM MgCl_2_, and 10mM NaHCO_3_ for 15min. The initial measurements of Rubisco activity were carried out in 0.1ml of reaction medium containing 5mM HEPES-NaOH (pH 8.0), 1mM NaHCO_3_, 2mM MgCl_2_, 0.25mM dithiothreitol (DTT), 0.1mM EDTA, 1U of glyceraldehyde 3-phosphate dehydrogenase (GAPDH), 0.5mM ATP, 0.015mM NADH_2_, 0.5mM phosphocreatine, 0.06mM RuBP, and 10 μl of extract. The change in absorbance at 340nm was monitored for 90 s. RCA activity was determined using a Rubisco Activase Assay Kit (Genmed Scientifics, Washington, DC, USA). FBPase activity was determined by monitoring the increase in *A*_340_ using an extinction coefficient of 6.2mM^–1^ cm^–1^ (Scheibe *et al.*, 1986). Total activity was assayed after the crude extract had been activated in a 0.1ml activation mixture containing 100mM DTT, 2mM FBP, 10mM MgCl_2_, and 0.1M TRIS-HCl (pH 8.0). The initial activity was assayed immediately after homogenization. The assay mixture consisted of 0.1M HEPES-NaOH (pH 8.0), 0.5mM Na_2_EDTA, 10mM MgCl_2_, 0.3mM NADP^+^, 0.6mM fructose-1,6-bisphosphate, 0.6U of glucose-6-phosphate dehydrogenase from baker’s yeast (Sigma, Santa Clara, CA, USA), 1.2U of glucoe phosphate isomerase from baker’s yeast (Sigma, Santa Clara, CA, USA), and 100 μl of enzyme extract in a final volume of 1ml.

### Measurements of glutathione contents and the activity of enzymes involved in the AsA–GSH cycle

For the measurement of reduced glutathione (GSH) and oxidized glutathione (GSSG), plant leaf tissue (0.3g) was homogenized in 2ml of 6% metaphosphoric acid containing 2mM EDTA and centrifuged at 4 °C for 10min at 12 000 *g*. After neutralization with 0.5M phosphate buffer (pH 7.5), 0.1ml of the supernatant was added to a reaction mixture containing 0.2mM NADPH, 100mM phosphate buffer (pH 7.5), 5mM EDTA, and 0.6mM 5,5’-dithio-bis (2-nitrobenzoic acid). The reaction was initiated by adding 3U of glutathione reductase (GR) and was monitored by measuring the changes in absorbance at 412nm for 1min. For the GSSG assay, GSH was masked by the addition of 40 μl of 2-vinylpyridine to the neutralized supernatant, whereas 40 μl of water was added for the total glutathione assay. The GSH concentration was obtained by subtracting the GSSG concentration from the total concentration (Rao and Ormrod, 1995).

To determine the enzymatic activities of proteins involved in the AsA–GSH cycle, leaf tissue (0.3g) was ground in 3ml of ice-cold buffer containing 25mM HEPES (pH 7.8), 0.2mM EDTA, 2mM ascorbic acid, and 2% PVP. The homogenates were centrifuged at 4 °C for 20min at 12 000 *g*, and the resulting supernatants were used to determine the enzymatic activity. The ascorbate peroxidase (APX) and dehydroascorbate reductase (DHAR) activities were evaluated by measuring the decrease in absorbance at 290nm and the increase in absorbance at 265nm, as described by Nakano and Asada (1981). Monodehydroascorbate reductase (MDAR) activity was measured using 1U of ascorbate oxidase, and the oxidation rate of NADH was followed at 340nm (Hossain *et al.*, 1984). GR activity was measured according to the method reported by Halliwell and Foyer (1976), which is based on the rate at which the absorbance of NADPH decreases at 340nm. All spectrophotometric analyses were conducted in a SHIMADZU UV-2410PC spectrophotometer (Shimadzu Corporation, Kyodo, Japan).

### Total RNA extraction and gene expression analysis

Total RNA was isolated from tomato leaves using the TRIZOL reagent (Sangon, Shanghai, China) according to the instructions supplied by the manufacturer. After extraction, the total RNA was dissolved in diethyl pyrocarbonate-treated water. The cDNA template for qRT-PCR was synthesized from 2 μg of total RNA using the ReverTra Ace qPCR RT Kit (Toyobo, Osaka, Japan).

For qRT-PCR analysis, PCR products were amplified in triplicate using iQ SYBR Green SuperMix (Bio-Rad, Hercules, CA, USA) in 25 μl qRT-PCR assays. PCR was performed using the iCycler iQ 96-well real-time PCR Detection System (Bio-Rad, Hercules, CA, USA), and the cycling conditions consisted of denaturation at 95 °C for 3min, followed by 40 cycles of denaturation at 95 °C for 30 s, annealing at 58 °C for 30 s, and extension at 72 °C for 30 s. The tomato *actin* gene was used as an internal control. Gene-specific primers were designed according to expressed sequence tag (EST) sequences and were employed for amplification as described in Supplementary Table S2 available at *JXB* online. Relative gene expression was calculated as described by Livak and Schmittgen (2001).

### Statistical analysis

The experimental design was a completely randomized block design with four replicates. Each replicate contained 10 plants. Statistical analysis of the bioassays was performed using the SAS statistical package. The differences between the treatment means were separated using Tukey’s test at a level of *P*<0.05.

## Results

### BRs-induced changes in chloroplast TRX transcripts in tomato

**Fig. 1.**
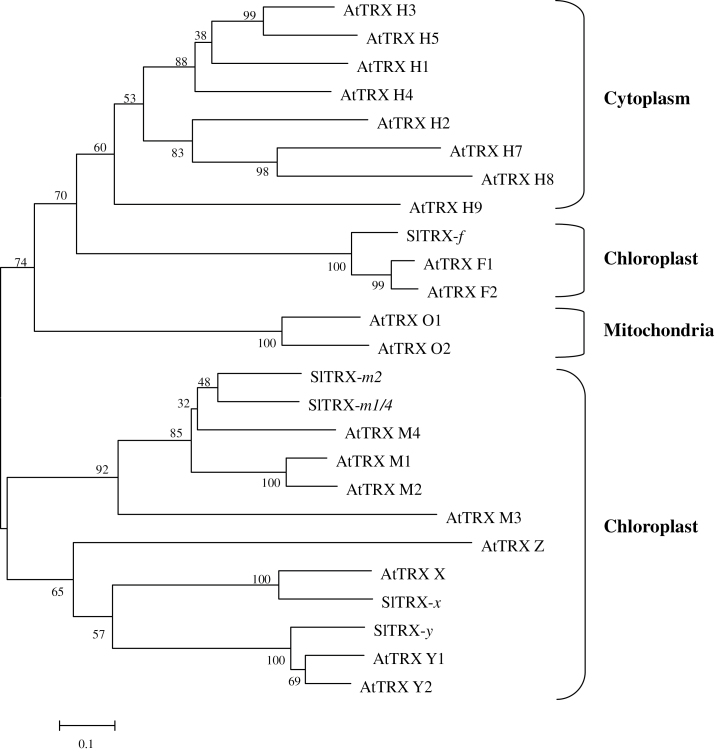
Phylogenetic tree of chloroplastic thioredoxins from *Solanum lycopersicum* (Sl) and those identified thioredoxins from *Arabidopsis* (At). The phylogenetic tree was constructed using MEGA 5 with the Neighbor–Joining method. Bootstrap values calculated from 1000 trials are shown at each node. The extent of divergence according to the scale (relative units) is indicated at the bottom. Predicted mature polypeptides lacking the putative transit peptide were employed for tree construction.

**Fig. 2.**
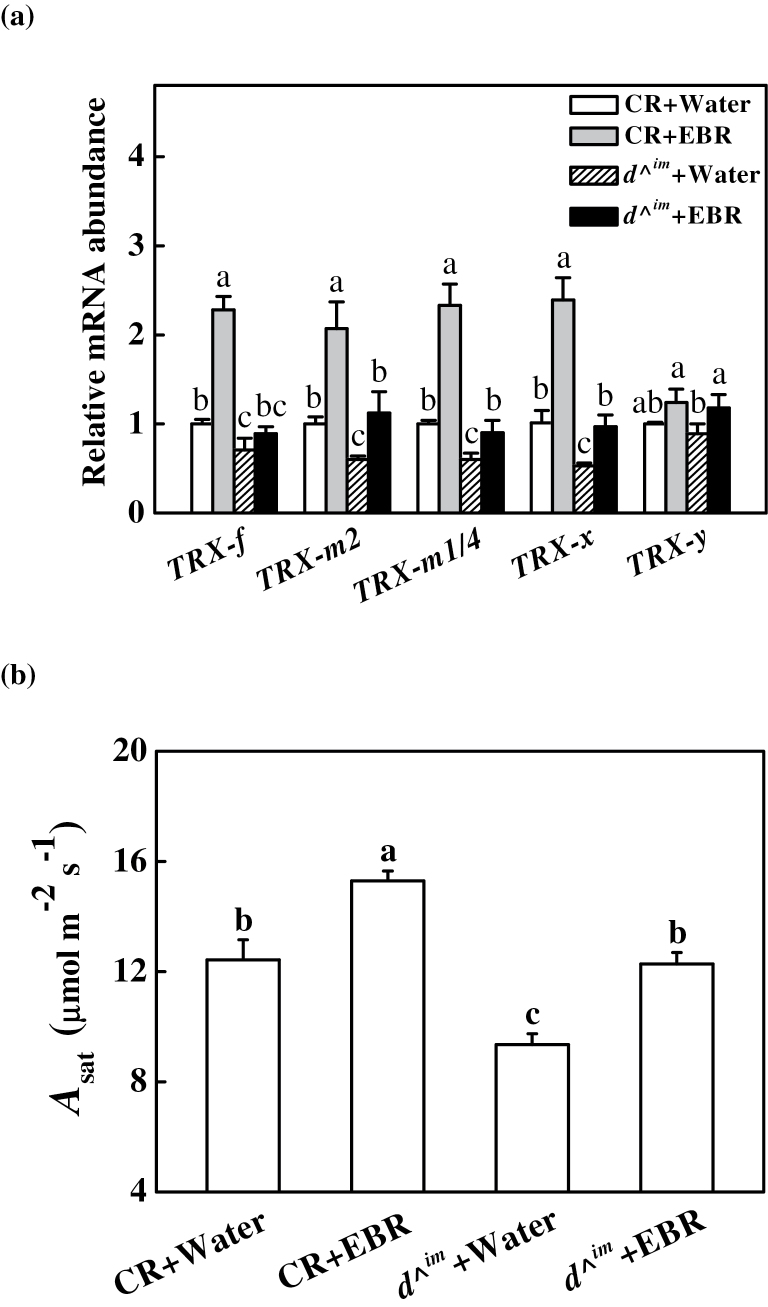
Effects of EBR on transcripts of chloroplastic *TRX* genes (a) and light-saturated rate of CO_2_ assimilation (*A*_sat_) in Condine Red (CR) and BRs-deficient *d* ^*^im*^ plants (b). *A*_sat_ was measured at 24h after EBR treatment in CR and *d* ^*^im*^ plants. Leaf samples were taken at 24h after EBR treatment for gene expression analysis. Relative gene expression for each *TRX* gene was calculated as the water-treated CR plants (control)=1. Data are the means of four replicates with SDs. Means followed by the same letter are not significantly different according to Tukey’s test (*P*<0.05).

A database (Tomato Genome Sequencing Project) search based on sequence similarity with the predicted chloroplast TRXs of *Arabidopsis* indicated five chloroplast TRX nucleotide sequences in *Solanum lycopersicum*: *TRX-f* (Solyc05g056300), *TRX-m2* (Solyc10g006970), *TRX-m1/4* (Solyc12g013810), *TRX-x* (Solyc01g008250), and *TRX-y* (Solyc04g071560). A phylogenetic tree built from the alignment of these five proteins with the previously identified *Arabidopsis* TRXs revealed the evolutionary distances between the sequences (Fig. 1). Among these sequences, SlTRX-*f*, -*x*, and -*y* showed 100% similarity to the sequences of AtTRX-*f*, -*x*, and -*y*, while SlTRX-*m*2 and -*m*1/4 showed low similarity to their orthologues in *Arabidopsis*. To examine how the transcription of these *TRX* genes is influenced by BRs, their transcript levels were determined following EBR treatment in CR and *d* ^*^im*^ plants. Transcript levels for *TRX-f*, *TRX-m2*, *TRX-m1/4*, and *TRX-x* were reduced by 30–40% in *d* ^*^im*^ plants compared with CR plants, respectively, with the exception of *TRX-y* (Fig. 2a). In contrast, *TRX-f*, *TRX-m2*, *TRX-m1/4*, and *TRX-x* transcript levels were upregulated ~1-fold following treatment with EBR in CR plants. Similarly, no significant change was observed in *TRX-y* transcript levels in the EBR-treated CR plants. Interestingly, the expression of those *TRX* genes was restored to a level similar to that in the water-treated CR plants when *d* ^*^im*^ plants were treated with EBR (Fig. 2a).

### The role of chloroplast TRXs in BRs-induced CO_2_ assimilation

BRs levels are closely related to CO_2_ assimilation in cucumber plants (Yu *et al.*, 2004; Xia *et al.*, 2009*a*). Here, it was found that the light-saturated rate of CO_2_ assimilation (*A*_sat_) was reduced by 24.7% in BRs-deficient *d* ^*^im*^ mutant plants compared with wild-type CR plants. However, exogenous application of 0.2 μM EBR increased the *A*_sat_ value in CR plants 24h after EBR application. Furthermore, exogenous EBR increased the *A*_sat_ of the *d* ^*^im*^ plants to the level of untreated CR plants (Fig. 2b).

**Fig. 3.**
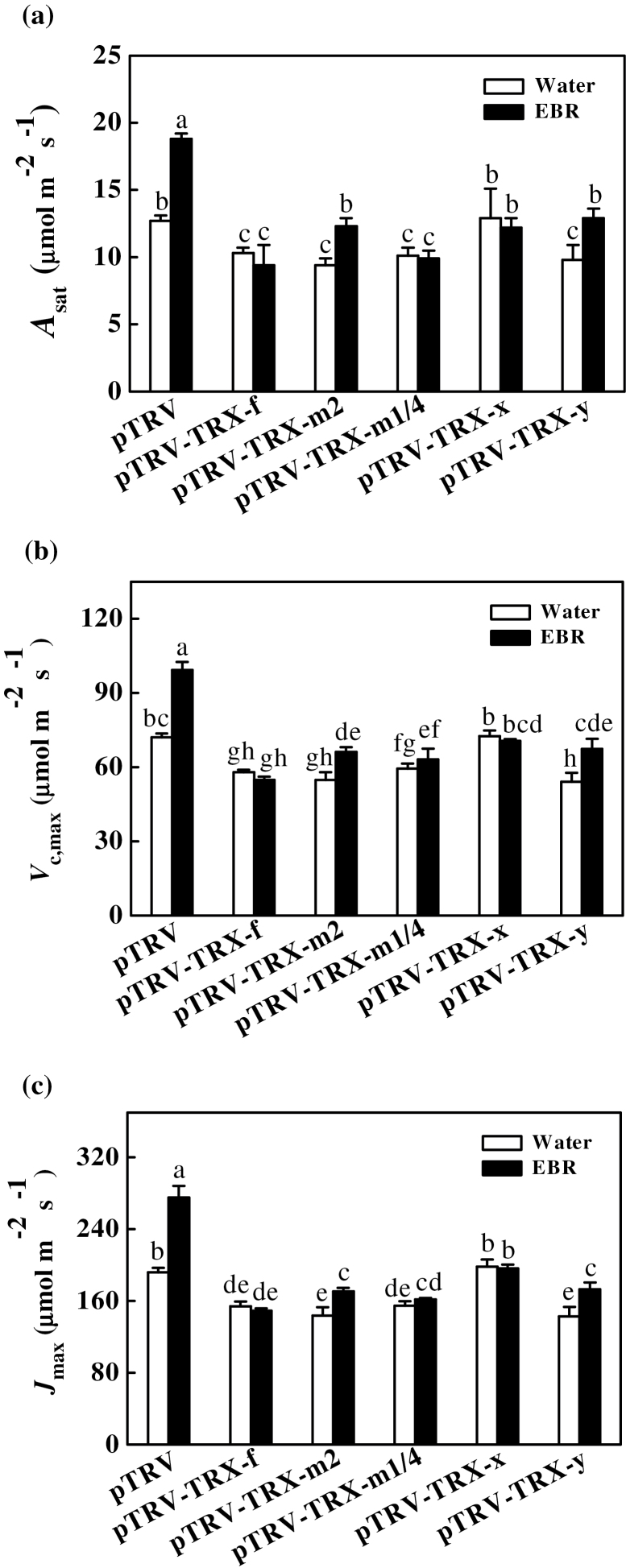
Changes in light-saturated rate of CO_2_ assimilation (*A*_sat_) (a), maximum Rubisco carboxylation rate (*V*_c,max_) (b), and maximum RuBP regeneration rate (*J*_max_) (c) for control and EBR-treated virus-induced gene silencing (VIGS) plants. Gas exchange measurements were carried out at 24h after EBR treatment. Data are the means of four replicates with SDs. Means followed by the same letter are not significantly different according to Tukey’s test (*P*<0.05).

To determine the role of these TRXs in BRs-induced CO_2_ assimilation, the five *TRX* genes were partially silenced individually using a VIGS method. Transcript analysis of the leaflets in the middle of the fifth fully expanded leaves revealed that the transcripts for these genes were reduced by 65–85% in the respective silenced plants (Supplementary Fig. S1 available at *JXB* online). To investigate the underlying molecular mechanisms of BRs-induced CO_2_ assimilation, the effect of EBR on CO_2_ assimilation were analysed in partially *TRX-f-*silenced (pTRV-*TRX-f*,), *TRX-m2*-silenced (pTRV-*TRX-m2*), *TRX-m1/4*-silenced (pTRV-*TRX-m1/4*), *TRX-x*-silenced (pTRV-*TRX-x*), and *TRX-y*-silenced (pTRV-*TRX-y*) plants. As shown in Fig. 3a, partially silencing the *TRX-f*, *TRX-m2*, *TRX-m1/4*, and *TRX-y* genes resulted in decreases in the *A*_sat_ of 18.9, 26.0, 20.5, and 22.8%, respectively, compared with the pTRV control plants. However, partially silencing *TRX-x* did not result in significant changes in *A*_sat_. The application of 0.2 μM EBR increased *A*_sat_ in the pTRV, pTRV-*TRX-m2*, and pTRV-*TRX-y* plants by 48.0, 30.9, and 31.6%, respectively, but had little effect on the pTRV-*TRX-f*, pTRV-*TRX-m1/4*, and pTRV-*TRX-x* plants. Similar to the observed changes in *A*_sat_, the *V*_c,max_ and the *J*_max_ values were significantly decreased in the pTRV-*TRX-f*, pTRV-*TRX-m2*, pTRV-*TRX-m1/4*, and pTRV-*TRX-y* plants but were not changed in the pTRV-*TRX-x* plants (Fig. 3b, c). Accordingly, exogenous application of EBR increased *V*_c,max_ and *J*_max_ only in the pTRV, pTRV-*TRX-m2*, and pTRV-*TRX-y* plants and not in the pTRV-*TRX-f*, pTRV-*TRX-m1/4,* and pTRV-*TRX-x* plants. All of these results indicated that chloroplastic TRX-*f*, TRX-*m*2, TRX-*m*1/4, and TRX-*y* are involved in the regulation of CO_2_ assimilation, whereas only TRX-*f* and TRX-*m*1/4 play a role in the EBR-induced increase in CO_2_ assimilation.

### TRX-induced changes in redox homeostasis influenced by BRs

**Fig. 4.**
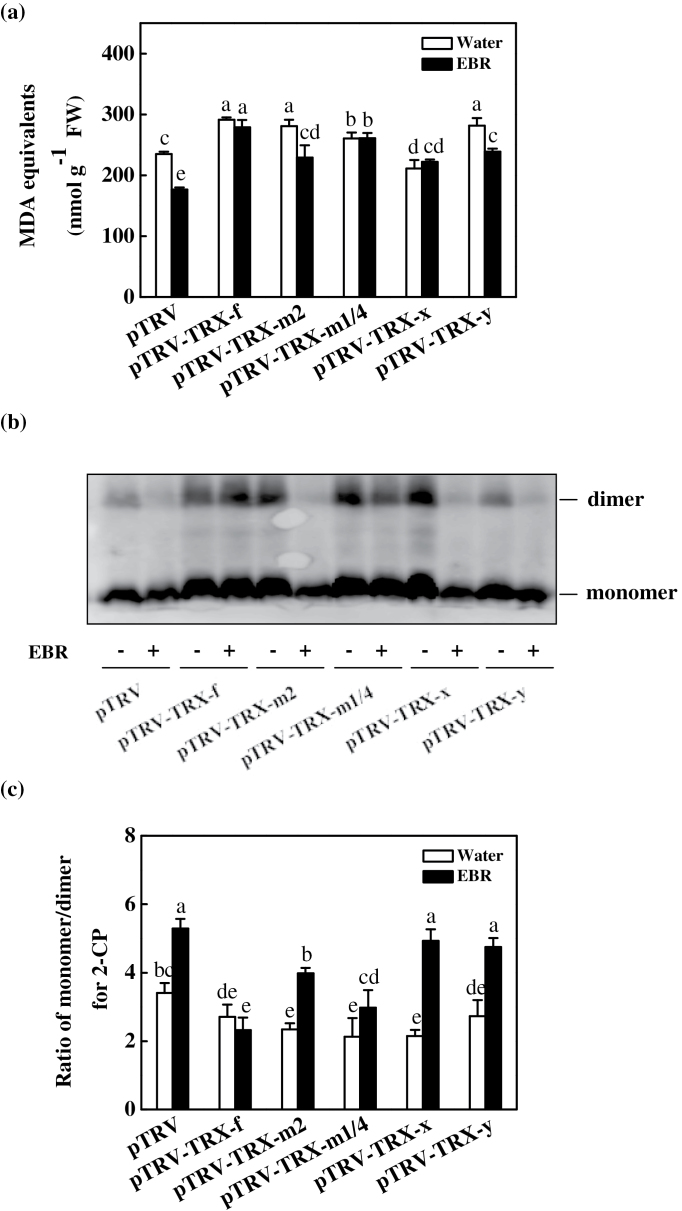
Changes in the lipid peroxidation and the redox state of 2-Cys peroxiredoxin (2-CP) protein in the leaves of virus-induced gene silencing (VIGS) plants as influenced by EBR application. (a) Changes in content of malondialdehyde (MDA) equivalents. (b) Changes in the redox state of 2-CP as investigated by non-reducing SDS–PAGE. The samples were separated by non-reducing SDS–PAGE and analysed in a western blot analysis with anti-2-CP. (c) The ratio of monomer/dimer for 2-CP from (b) as quantified by Quantity One. Leaf samples were taken at 24h after EBR treatment. Data are the means of four replicates with SDs. Means followed by the same letter are not significantly different according to Tukey’s test (*P*<0.05).

MDA is a useful indicator of reactive oxygen species (ROS)-induced lipid peroxidation in plants. Increased MDA accumulation was detected in the partially *TRX-f*-, *TRX-m2*-, *TRX-m1/4*-, and *TRX-y*-silenced plants, but not in the partially *TRX-x*-silenced plants. EBR treatment reduced the MDA content only in the pTRV, pTRV-*TRX-m2*, and pTRV-*TRX-y* plants and had no effect on the pTRV-*TRX-f*, pTRV-*TRX-m1/4*, and pTRV-*TRX-x* plants (Fig. 4a). Western blot analysis showed that 2-CP was mostly present in a reduced state (monomer), while ~22.7% was in an oxidized state (dimer) in the pTRV plants. In contrast, partial silencing of the TRXs resulted in an increase in the levels of oxidized 2-CP in the leaves, while the ratio of 2-CP monomers/2-CP dimers decreased by 20.5, 31.4, 37.5, 37.0, and 19.9% in the pTRV-*TRX-f*, pTRV-*TRX-m2*, pTRV-*TRX-m1/4*, pTRV-*TRX-x*, and pTRV-*TRX-y* plants, respectively, compared with the pTRV control plants. The application of 0.2 μM EBR increased the ratio of reduced 2-CP/oxidized 2-CP in the pTRV, pTRV-*TRX-m2*, pTRV-*TRX-x*, and pTRV-*TRX-y* plants by 55.1, 70.1, 129.3, and 74.0%, respectively, but had little effect on the pTRV-*TRX-f* and pTRV-*TRX-m1/4* plants (Fig. 4b, c).

### Involvement of glutathione redox homeostasis in BRs-induced CO_2_ assimilation

**Fig. 5.**
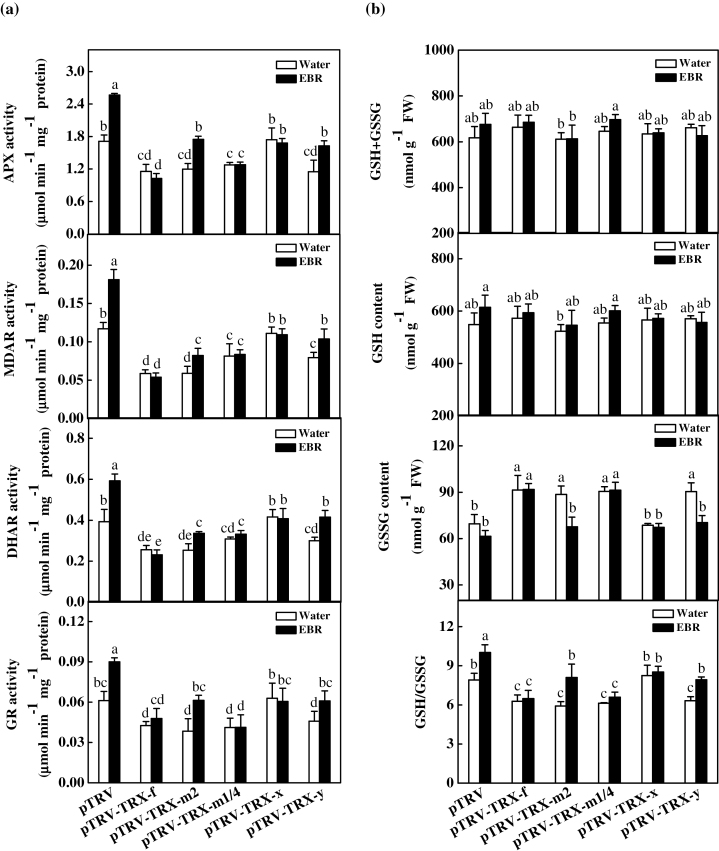
Changes in the activity of key enzymes involved in the AsA–GSH cycle and glutathione redox status for control and EBR-treated virus-induced gene silencing (VIGS) plants. Leaf samples were taken at 24h after EBR treatment. Data are the means of four replicates with SDs. Means followed by the same letter are not significantly different according to Tukey’s test (*P*<0.05).

TRX can regulate the activity of the AsA–GSH cycle, which plays a critical role in maintaining the cellular redox status. In this study, the changes in the activities of APX, MDAR, DHAR, and GR, which are four important enzymes involved in the AsA–GSH cycle, were analysed. As shown in Fig. 5a, partial silencing of *TRX-f*, *TRX-m2*, *TRX-m1/4*, and *TRX-y* decreased the activity of APX, MDAR, DHAR, and GR. However, no significant effects on the activities of these enzymes were observed in the pTRV-*TRX-x* plants. EBR treatment significantly increased the activity of the AsA–GSH cycle in the pTRV control, pTRV-*TRX-m2*, and pTRV-*TRX-y* plants, but had no effect in the pTRV-*TRX-f*, pTRV-*TRX-m1/4*, and pTRV-*TRX-x* plants. To analyse the EBR-induced changes in glutathione redox homeostasis further, the effects of EBR on the contents of GSH and GSSG and their ratios in the pTRV and various pTRV-*TRX* plants were compared. There was generally little change in GSH content observed in the pTRV and partially *TRX*-silenced plants (Fig. 5b). However, partial silencing of *TRX-f*, *TRX-m2*, *TRX-m1/4*, and *TRX-y* resulted in 31.5, 27.5, 30.2, and 30.1% increases in the GSSG content, respectively, leading to a decrease in the GSH/GSSG ratio. In contrast, EBR induced a slight increase in GSH content and decrease in GSSG, resulting in a significant increase in the GSH/GSSG ratio. Similar to the observed changes in the activities of the AsA–GSH cycle enzymes, the EBR-induced increases in the glutathione pool and GSH/GSSG ratio were abolished by partially silencing *TRX-f* and *TRX-m1/4* but not by the silencing of other TRXs (Fig. 5a, b).

### Gene expression and enzyme activities involved in the Benson–Calvin cycle influenced by the TRXs and BRs

**Fig. 6.**
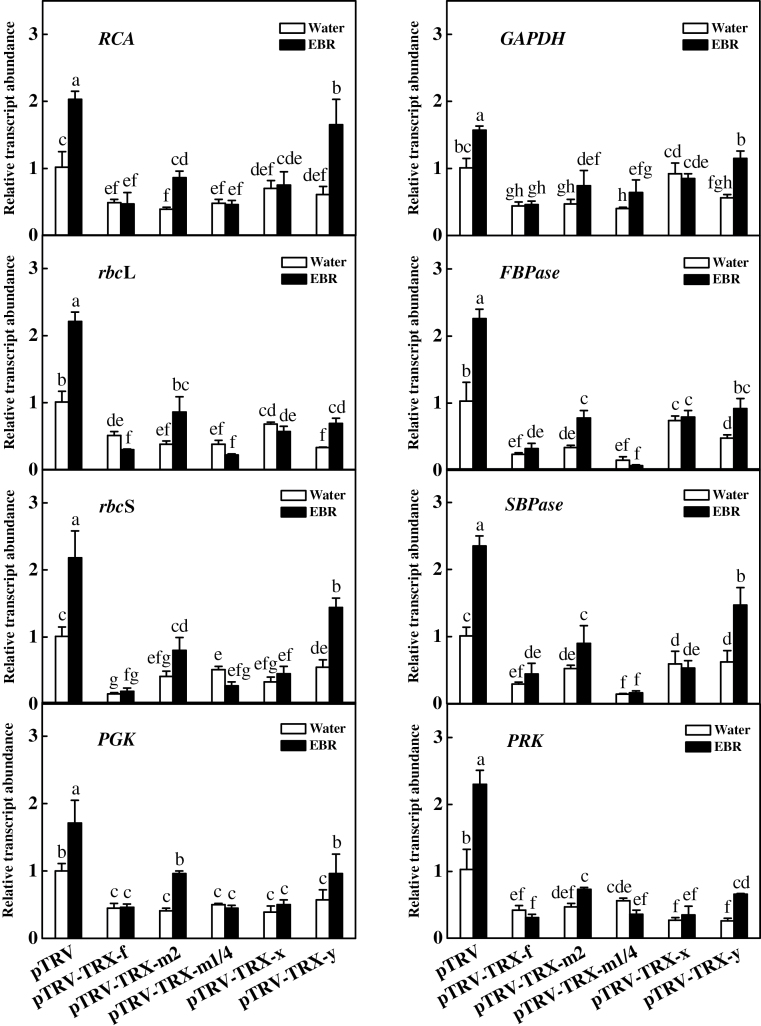
Changes in the expression of Benson–Calvin cycle-related genes for control and EBR-treated virus-induced gene silencing (VIGS) plants. Leaf samples were taken at 24h after EBR treatment. Data are the means of four replicates with SDs. Means followed by the same letter are not significantly different according to Tukey’s test (*P*<0.05).

Previously, it was reported that BRs-induced CO_2_ assimilation is associated with increased expression of photosynthetic genes in cucumber plants (Yu *et al.*, 2004; Xia *et al.*, 2009*a*). In this study, the transcript levels of eight Benson-Calvin cycle-related genes in the leaves of plants lacking different chloroplastic *TRX* genes were analysed. These tested photosynthesis-related genes included the genes encoding Rubisco activase (*RCA*), Rubisco large subunit (*rbc*L), Rubisco small subunit (*rbc*S), glycerate-3-phosphate kinase (*PGK*), glyceraldehyde-3-phosphate dehydrogenase (*GAPDH*), fructose-1,6-bisphosphatase (*FBPase*), sedoheptulose-1,7-bisphosphatase (*SBPase*), and ribulose-5-phosphate kinase (*PRK*). As shown in Fig. 6, the transcripts of these genes were all up-regulated upon EBR treatment in the pTRV plants. In contrast, partially silencing the chloroplastic *TRX* genes resulted in down-regulation of these photosynthesis-related genes. Importantly, EBR-induced transcripts were detected only in the pTRV-*TRX-m2* and pTRV-*TRX-y* plants and not in the other plants.

**Fig. 7.**
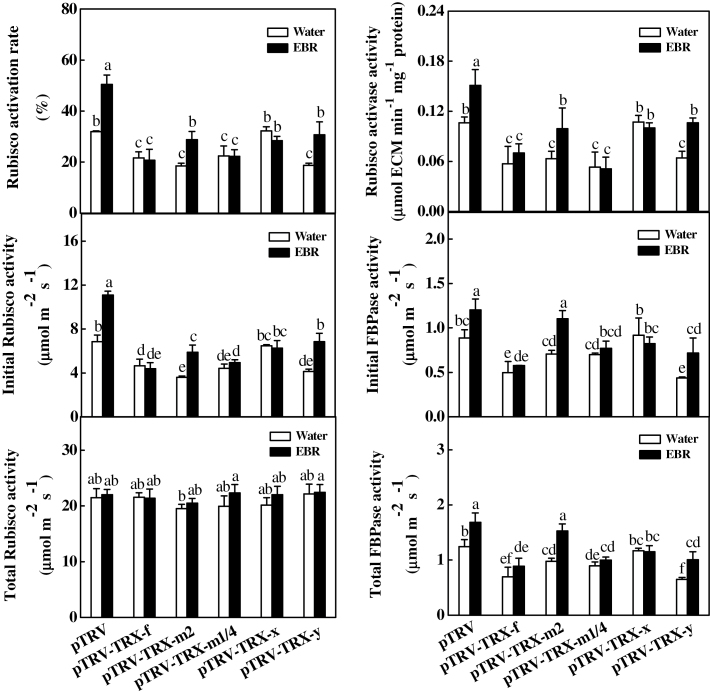
Changes in the activity of Rubisco activase, Rubisco, and FBPase for control and EBR-treated virus-induced gene silencing (VIGS) plants. Leaf samples were taken at 24h after EBR treatment. Data are the means of four replicates with SDs. Means followed by the same letter are not significantly different according to Tukey’s test (*P*<0.05).

BRs levels are closely related to the activation of a subset of enzymes involved in the Benson–Calvin cycle, particularly the redox-sensitive enzymes (Yu *et al.*, 2004; Xia *et al.*, 2009*a*). Here, the changes in the activities of RCA, Rubisco, and FBPase were analysed in VIGS plants with and without exogenous application of EBR. As shown in Fig. 7, partial silencing of these genes did not result in significant changes in total Rubisco activity but significantly decreased the initial Rubisco, initial FBPase, and Rubisco activase activities as well as the Rubisco activation rate, except in the pTRV-*TRX-x* plants, where these parameters were not significantly altered. For example, initial Rubisco activities decreased by 31.9, 47.4, 35.6, and 39.6%, while the Rubisco activation rate decreased by 32.1, 42.0, 29.8, and 41.3% in the pTRV-*TRX-f*, pTRV-*TRX-m2*, pTRV-*TRX-m1/4*, and pTRV-*TRX-y* plants, respectively, compared with the pTRV plants. Furthermore, exogenous EBR application increased the activity or the activation rate only in the pTRV-*TRX-m2* and pTRV-*TRX-y* plants. The activity and the activation rates of these enzymes in the EBR-treated VIGS plants were much lower than in the pTRV control plants.

## Discussion

### TRX-*f*, TRX-*m*2, TRX-*m*1/4, and TRX-*y* are involved in the regulation of cellular redox homeostasis via the AsA–GSH cycle and 2-CP

TRXs are involved in the regulation of ROS metabolism and CO_2_ assimilation. The results of the present study demonstrated that TRXs can differentially regulate oxidative stress in tomato plants. In rice, *Ostrxm* RNAi plants exhibit increased H_2_O_2_ accumulation in the leaves (Chi *et al.*, 2008). Histochemical staining demonstrated that more O_2_^·−^ and H_2_O_2_ accumulates in the leaves of the VIGS-*TRX*-*f*/*TRX*-*m* pea plants compared with control plants, confirming that TRX-*f* and TRX-*m* are important for the metabolic balance of ROS in plant cells (Luo *et al.*, 2012). Here, it was found that partially silencing *TRX-f*, *TRX-m2*, *TRX-m1/4*, or *TRX-y* resulted in increased oxidative stress, as indicated by the increased MDA content in the leaves, while partially silencing *TRX-x* did not induce significant changes in the MDA content of the leaves (Fig. 4a). These findings revealed that chloroplastic TRX-*f*, TRX-*m*2, and TRX-*m*1/4 as well as TRX-*y* are involved in ROS metabolism. It seems likely that TRX-*x* plays an insignificant role in ROS metabolism, as partially silencing *TRX-x* did not induce oxidative stress in the leaves.

ROS accumulation is closely related to the ROS scavenging capacity of cells, which is largely dependent on antioxidant enzymes, such as APX, MDAR, DHAR, and GR, and non-enzymatic antioxidants, such as AsA and GSH. Proteomic studies in *Arabidopsis* have determined that several key enzymes involved in the AsA–GSH cycle, such as GR, are redox sensitive (Ströher and Dietz, 2008; Wang *et al.*, 2012), and biochemical and genetic assays have identified the NADPH-dependent thioredoxin system as a backup system for GR1 (Marty *et al.*, 2009). Until now, few studies have investigated the role of TRXs in the regulation of antioxidant activity in plants. Here, it is demonstrated that partially silencing the *TRX-f*, *TRX-m2*, T*RX-m1/4*, and *TRX-y* genes resulted in decreased activity of the enzymes APX, MDAR, DHAR, and GR, while partially silencing the *TRX-x* gene did not induce significant changes in the activity of these enzymes (Fig. 5a). Additionally, partially silencing *TRX-f*, *TRX-m2*, *TRX-m1/4*, and *TRX-y* did not alter the total glutathione (GSH+GSSG) or GSH content but did induce an increase in the GSSG content, leading to a decrease in the GSH/GSSG ratio (Fig. 5b). These results indicated that TRX-*f*, TRX-*m*2, TRX-*m*1/4, and TRX-*y* do not influence GSH biosynthesis but affect glutathione homeostasis by regulating GR activity, as observed in both the glutathione-deficient *cad2* and GR-deficient *gr1 Arabidopsis* mutants (Meyer *et al.*, 2007; Marty *et al.*, 2009). However, such an effect was not observed in the pTRV-*TRX-x* plants. All of these results suggested that the enzymes involved in the AsA–GSH cycle are largely modified by TRX-*f*, TRX-*m*2, TRX-*m*1/4, and TRX-*y*, and that the oxidative stress induced in partially *TRX-f*, *TRX-m2*, *TRX-m1/4*, and *TRX-y*-silenced plants is at least in part attributable to the reduced activity of the antioxidant enzymes. The finding that the cellular glutathione redox status could be regulated by chloroplastic TRXs further revealed the existence of cross-talk between the TRX system and the glutathione system.

In addition to the AsA–GSH cycle, peroxiredoxin, which is activated by TRX, acts as an alternative pathway to remove H_2_O_2_ from chloroplasts (Foyer and Shigeoka, 2011). The 2-CP enzyme exhibits broad substrate specificity, showing activity toward both hydrogen peroxides and complex alkyl hydroperoxides. During the peroxide reduction reaction, 2-CP is alternatively oxidized and reduced as it catalyses the electron flow from an electron donor to peroxide. *In vitro* tests have demonstrated that TRX-*x* is by far the most efficient in *Arabidopsis* (Collin *et al.*, 2003). However, only the oxidized dimeric form of 2-CP was detected in the *Ostrxm* RNAi plants, suggesting that Ostrxm could also regulate the catalytic activity of 2-CP by reducing redox-active cysteine residues in plants (Chi *et al.*, 2008). The present study revealed increased accumulation of oxidized 2-CP in all of the partially *TRX*-silenced plants, with the partial silencing of *TRX-x*, *TRX-m1/4*, and *TRX-m2* being more significant, suggesting that these TRXs could differentially regulate 2-CP (Fig. 4b, c).

### TRX-*f* and TRX-*m*1/4 play important roles in BRs-induced changes in cellular redox homeostasis

As observed in previous research, foliar application of EBR relieved oxidative stress (Xia *et al.*, 2009*b*). In addition to increasing the activity of the AsA–GSH cycle, EBR also induced an increase in the ratio of the reduced monomers to the oxidized dimers of 2-CP (Fig. 4c). Importantly, partially silencing *TRX-f* and *TRX-m1/4* compromised the EBR-induced decrease in MDA content and increase in the activity of antioxidant enzymes, as well as the associated changes in glutathione metabolism (Figs 4a, 5a, b), suggesting that EBR partially alleviates oxidative stress by regulating TRX-*f* and TRX-*m*1/4.

It was found that the TRXs are actively involved in the regulation of photosynthesis-related gene transcripts, enzyme activity, and, ultimately, the CO_2_ assimilation capacity. *Ostrxm* plants exhibit abnormal chloroplast development and growth inhibition in rice plants, displaying decreased levels of several chloroplast proteins that are critical for photosynthesis and biogenesis (Chi *et al.*, 2008). Silencing *TRX-f* and *TRX-m* (VIGS-TRX-*f*/TRX-*m*) in pea plants results in a significant reduction in Mg chelatase activity and the 5-aminolaevulinic acid synthesizing capacity as well as decreased transcript levels of *RBCS* and chlorophyll biosynthesis-related genes and a decreased photosynthetic capacity (Luo *et al.*, 2012). In the present study, it was found that partially silencing the chloroplastic TRXs, except for TRX-*x*, resulted in a reduced photosynthetic capacity in tomato leaves (Fig. 3), supporting the hypothesis that TRX-*f*, TRX-*m*2, TRX-*m*1/4, and TRX-*y* are actively involved in the regulation of CO_2_ assimilation. In *Arabidopsis*, the maximal (*F*_v_/*F*_m_) and effective quantum yields of photosystem II (PSII; Ф_PSII_) in the *trx f1.1* and *trx f1.2* mutant plants are not different from those in wild-type plants (Thormählen *et al.*, 2013). In agreement with this finding, it was observed that only the pTRV-*TRX-m1/4* plants showed a decrease in *F*_v_/*F*_m_, by 15.4% (Supplementary Fig. S2 available at *JXB* online). All of these results suggested that the TRXs do not affect CO_2_ assimilation by modulating PSII electron transport.

### TRX-*f* and TRX-*m*1/4 play important roles in BRs-induced changes in CO_2_ assimilation

Previous studies have shown that chloroplastic TRXs participate in the regulation of the Calvin cycle and associated processes (Balmer *et al.*, 2003). In addition to the down-regulation of genes involved in the Benson–Calvin cycle, partially silencing *TRX-f*, *TRX-m2*, *TRX-m1/4*, and *TRX-y* also resulted in a significant decrease in the initial Rubisco and FBPase activities as well as RCA activity (Fig. 7). An *in vitro* assay revealed that AtTRX-*f* is able to activate FBPase and NADP-MDH most efficiently, followed by AtTRX-*m*1, AtTRX-*m*2, and AtTRX-*m*4, whereas AtTRX-*m*3, AtTRX-*x*, and AtTRX-*y* cannot efficiently activate NADP-MDH from sorghum or from *Chlamydomonas* (Collin *et al.*, 2003, 2004; Lemaire *et al*., 2003, 2005). The simultaneous decrease in *V*_c,max_ and initial Rubisco activity, with little change in total Rubisco activity, indicates that chloroplastic TRX-*f*, TRX-*m*2, TRX-*m*1/4, and TRX-*y* mainly regulate the activation state of Rubisco via the action of Rubisco activase (Figs 3b, 7). The regeneration of RuBP is dependent on both the photosynthetic electron transport chain and the enzymes downstream of Rubisco in the Calvin cycle (Long *et al.*, 2006). In addition to the decrease in RCA activity, initial FBPase activity was also decreased in the plants in which *TRX-f*, *TRX-m2*, *TRX-m1/4*, and *TRX-y* were partially silenced (Fig. 7). Accordingly, the lack of direct activation of RCA or FBPase was involved in the decrease in *J*_max_. Interestingly, the observed changes in the activities of these enzymes were in agreement with cellular glutathione homoeostasis (Fig. 5b). In a previous study, it was found that several redox-sensitive Benson–Calvin cycle enzymes, such as RCA, can undergo disulphide bond interchange, leading to changes in the activities of these enzymes (Jiang *et al.*, 2012*b*). It is likely that the oxidized cellular environment induced by the partial silencing of *TRX-f*, *TRX-m2*, *TRX-m1/4*, and *TRX-y* can directly modify the structure of these redox-sensitive enzymes.

Previously, it was found that inhibition of the biosynthesis of BRs using an inhibitor decreased CO_2_ assimilation, while exogenously applied BRs increased CO_2_ assimilation in cucumber plants (Yu *et al.*, 2004; Xia *et al.*, 2009*a*). Here, it was demonstrated that exogenous EBR application resulted in enhanced CO_2_ assimilation in tomato plants, while BRs-deficient *d* ^*^im*^ mutant plants exhibited decreased CO_2_ assimilation compared with wild-type CR plants (Fig. 2b), providing further evidence that BRs levels are closely related to the CO_2_ assimilation capacity of plants.

BRs can differentially induce *TRX* transcripts in *Arabidopsis* and rice (Müssig *et al.*, 2002; Goda *et al.*, 2004; Wu *et al.*, 2008). In the present study, it was found that BRs were able to up-regulate *TRX-f*, *TRX-m2*, *TRX-m1/4*, and *TRX-x* but did not affect the transcription of *TRX-y* (Fig. 2a). In contrast, partially silencing *TRX-x* did not induce any changes in CO_2_ assimilation, the activities of antioxidant enzymes, redox homeostasis, or the expression and activities of photosynthesis-related genes and enzymes (Figs 3a, 5a, 5b, 6, 7), suggesting that TRX-*f*, TRX-*m*2, and TRX-*m*1/4 are potentially involved in the BRs-induced increase in CO_2_ assimilation. However, the partial silencing of *TRX-f*, *TRX-m2*, and *TRX-m1/4* resulted in decreases in CO_2_ assimilation, the expression of Benson–Calvin cycle-related genes, and the activities of associated enzymes, while EBR induced these changes only in pTRV-*TRX-m2* plants and not in pTRV-*TRX-f* or pTRV-*TRX-m1/4* plants (Figs 3a, 6, 7). These results suggest that TRX-*f* and TRX-*m*1/4 are the TRXs responsible for BRs-induced CO_2_ assimilation.

**Fig. 8.**
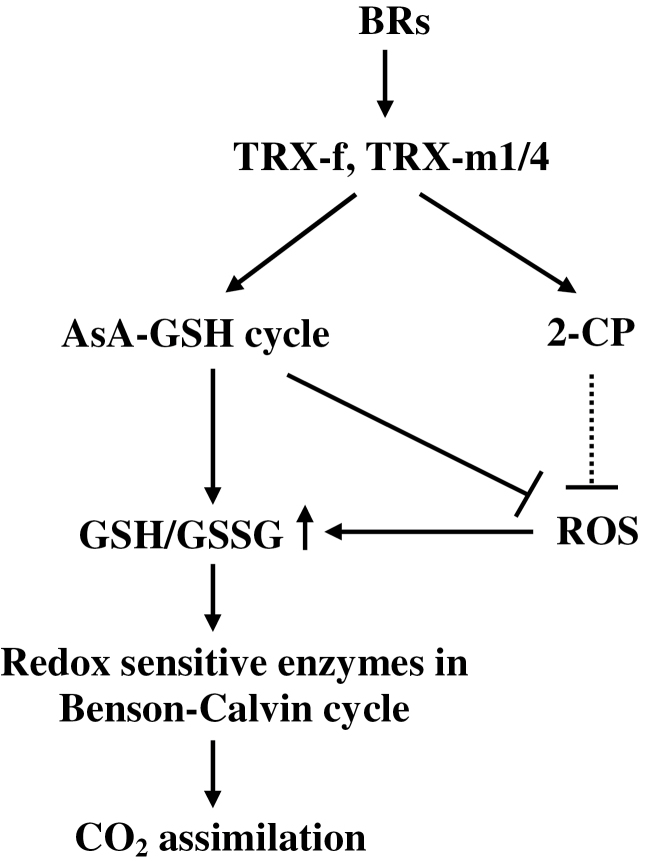
A proposed model for BRs-induced increase in CO_2_ assimilation in tomato plants.

Many processes involved in plant growth, development, and metabolism are under the tight control of the TRXs (Schürmann and Buchanan, 2008). The results of this study demonstrated that the chloroplastic TRXs of tomato play different roles in cellular redox homeostasis and CO_2_ assimilation, and BRs regulate cellular redox homeostasis and CO_2_ assimilation in a TRX-*f*- and TRX-*m*1/4-dependent manner (Fig. 8). Among the examined TRXs, TRX-*f*, TRX-*m*2, TRX-*m*1/4, and TRX-*y* were all able to activate the antioxidant system by up-regulating the AsA–GSH cycle and the Benson–Calvin cycle, while TRX-*x* is not involved in the regulation of these processes. In addition to their direct effects on gene transcription, BRs also increased the antioxidant capacity, leading to a reduced cellular environment, which could directly reduce specific disulphide bridges in photosynthesis-related redox-sensitive enzymes, leading to the activation of these enzymes and therefore playing an important role in the regulation of normal photosynthesis.

## Supplementary data

Supplementary data are available at *JXB* online.

Figure S1. Relative mRNA abundance of *TRX-f*, *TRX-m2*, *TRX-m1/4*, *TRX-x*, and *TRX-y* in respective virus-induced gene silencing (VIGS) plants.

Figure S2. Changes in maximum quantum yield of PSII (*F*_v_/*F*_m_) in the leaves of pTRV and various partially *TRX*-silenced plants.

Table S1. PCR primers designed for vector construction.

Table S2. Gene-specific primers designed for qRT-PCR.

Supplementary Data

## References

[CIT0001] Alkhalfioui F Renard M Vensel WH Wong J Tanaka CK Hurkman WJ Buchanan BB Montrichard F 2007 Thioredoxin-linked proteins are reduced during germination of *Medicago truncatula* seeds. Plant Physiology 144, 1559–1579 17513483 10.1104/pp.107.098103 PMC1914137

[CIT0002] Arsova B Hoja U Wimmelbacher M Greiner E Ustün S Melzer M Petersen K Lein W Börnke F 2010 Plastidial thioredoxin *z* interacts with two fructokinase-like proteins in a thiol-dependent manner: evidence for an essential role in chloroplast development in *Arabidopsis* and *Nicotiana benthamiana* . The Plant Cell 22, 1498–1515 20511297 10.1105/tpc.109.071001 PMC2899873

[CIT0003] Balmer Y Koller A del Val G Manieri W Schürmann P Buchanan BB 2003 Proteomics gives insight into the regulatory function of chloroplast thioredoxins. Proceedings of the National Academy of Sciences, USA 100, 370–375 10.1073/pnas.232703799 PMC140980 12509500

[CIT0004] Balmer Y Vensel WH Cai N Manieri W Schürmann P Hurkman WJ Buchanan BB 2006 A complete ferredoxin/thioredoxin system regulates fundamental processes in amyloplasts. Proceedings of the National Academy of Sciences, USA 103, 2988–2993 10.1073/pnas.0511040103 PMC1413819 16481623

[CIT0005] Balmer Y Vensel WH Tanaka CK 2004 Thioredoxin links redox to the regulation of fundamental processes of plant mitochondria. Proceedings of the National Academy of Sciences, USA 101, 2642–2647 10.1073/pnas.0308583101 PMC357003 14983062

[CIT0006] Bartoli CG Casalongué CA Simontacchi M Marquez-Garcia B Foyer CH 2013 Interactions between hormone and redox signalling pathways in the control of growth and cross tolerance to stress. Environmental and Experimental Botany 94, 73–88

[CIT0007] Baumann U Juttner J 2002 Plant thioredoxins: the multiplicity conundrum. Cellular and Molecular Life Sciences 59, 1042–1057 12169016 10.1007/s00018-002-8485-8 PMC11337411

[CIT0008] Bishop GJ Nomura T Yokota T Harrison K Noguchi T Fujioka S Takatsuto S Jones JDG Kamiya Y 1999 The tomato DWARF enzyme catalyses C-6 oxidation in brassinosteroid biosynthesis. Proceedings of the National Academy of Sciences, USA 96, 1761–1766 10.1073/pnas.96.4.1761 PMC15587 9990098

[CIT0009] Buchanan BB Balmer Y 2005 Redox regulation: a broadening horizon. Annual Review of Plant Biology 56, 187–220 10.1146/annurev.arplant.56.032604.144246 15862094

[CIT0010] Chi YH Moon JC Park JH Kim H-S Zulfugarov IS Fanata WI Jang HH Lee JR Lee YM Kim ST 2008 Abnormal chloroplast development and growth inhibition in rice thioredoxin *m* knock-down plants. Plant Physiology 148, 808–817 18723667 10.1104/pp.108.123547 PMC2556838

[CIT0011] Clouse SD Sasse JM 1998 Brassinosteroids: essential regulators of plant growth and development. Annual Review of Plant Physiology and Plant Molecular Biology 49, 427–451 10.1146/annurev.arplant.49.1.427 15012241

[CIT0012] Collin V Issakidis-Bourguet E Marchand C Hirasawa M Lancelin JM Knaff DB Miginiac-Maslow M 2003 The *Arabidopsis* plastidial thioredoxins: new functions and new insights into specificity. Journal of Biological Chemistry 278, 23747–23752 12707279 10.1074/jbc.M302077200

[CIT0013] Collin V Lamkemeyer P Miginiac-Maslow M Hirasawa M Knaff DB Dietz KJ Issakidis-Bourguet E 2004 Characterization of plastidial thioredoxins from *Arabidopsis* belonging to the new *y*-type. Plant Physiology 136, 4088–4095 15531707 10.1104/pp.104.052233 PMC535839

[CIT0014] Cui JX Zhou YH Ding JG Xia XJ Shi K Chen SC Asami T Chen Z Yu JQ 2011 Role of nitric oxide in hydrogen peroxide-dependent induction of abiotic stress tolerance by brassinosteroids in cucumber. Plant, Cell and Environment 34, 347–358 10.1111/j.1365-3040.2010.02248.x 21054437

[CIT0015] Ekengren SK Liu YL Schiff M Dinesh-Kumar SP Martin GB 2003 Two MAPK cascades, NPR1, and TGA transcription factors play a role in Pto-mediated disease resistance in tomato. The Plant Journal 36, 905–917 14675454 10.1046/j.1365-313x.2003.01944.x

[CIT0016] Ethier GJ Livingston NJ 2004 On the need to incorporate sensitivity to CO_2_ transfer conductance into the Farquhar–von Caemmerer–Berry leaf photosynthesis model. Plant, Cell and Environment 27, 137–153

[CIT0017] Foyer CH Shigeoka S 2011 Understanding oxidative stress and antioxidant functions to enhance photosynthesis. Plant Physiology 155, 93–100 21045124 10.1104/pp.110.166181 PMC3075779

[CIT0018] Geck MK Larimer FW Hartman FC 1996 Identification of residues of spinach thioredoxin *f* that influence interactions with target enzymes. Journal of Biological Chemistry 271, 24736–24740 8798742 10.1074/jbc.271.40.24736

[CIT0019] Goda H Sawa S Asami T Fujioka S Shimada Y Yoshida S 2004 Comprehensive comparison of auxin-regulated and brassinosteroid-regulated genes in *Arabidopsis* . Plant Physiology 134, 1555–1573 15047898 10.1104/pp.103.034736 PMC419831

[CIT0020] Halliwell B Foyer CH 1976 Ascorbic acid, metal-ions and the superoxide radical. Biochemistry Journal 155, 697–700 10.1042/bj1550697 PMC1172894 182136

[CIT0021] Hodges DM DeLong JM Forney CF Prange RK 1999 Improving the thiobarbituric acid-reactive-substances assay for estimating lipid peroxidation in plant tissues containing anthocyanin and other interfering compounds. Planta 207, 604–611 10.1007/s00425-017-2699-3 28456836

[CIT0022] Hodges M Miginiac-Maslow M Decottignies P Jacquot JP Stein M Lepiniec L Cretin C Gadal P 1994 Purification and characterization of pea thioredoxin f expressed in *Escherichia coli* . Plant Molecular Biology 26, 225–234 7948872 10.1007/BF00039534

[CIT0023] Hossain M Nakano Y Asada K 1984 Monodehydroascorbate reductase in spinach-chloroplast and its participation in regeneration of ascorbate for scavenging hydrogen-peroxide. Plant and Cell Physiology 25, 385–395

[CIT0024] Jacquot JP Gelhaye E Rouhier N Corbier C Didierjean C Aubry A 2002 Thioredoxins and related proteins in photosynthetic organisms: molecular basis for thiol dependent regulation. Biochemical Pharmacology 64, 1065–1069 12213606 10.1016/s0006-2952(02)01177-2

[CIT0025] Jacquot JP Lancelin JM Meyer Y 1997 Thioredoxins: structure and function in plant cells. New Phytologist 136, 543–570 10.1046/j.1469-8137.1997.00784.x 33863109

[CIT0026] Jiang YP Cheng F Zhou YH Xia XJ Mao WH Shi K Chen ZX Yu JQ 2012 *a* Cellular glutathione redox homeostasis plays an important role in the brassinosteroid-induced increase in CO_2_ assimilation in *Cucumis sativus* . New Phytologist 194, 932–943 22432590 10.1111/j.1469-8137.2012.04111.x

[CIT0027] Jiang YP Cheng F Zhou YH Xia XJ Mao WH Shi K Chen ZX Yu JQ 2012 *b* Brassinosteroid-induced CO_2_ assimilation is associated with increased stability of redox-sensitive photosynthetic enzymes in the chloroplasts in cucumber plants. Biochemical and Biophysical Research Communications 426, 390–394 22960180 10.1016/j.bbrc.2012.08.100

[CIT0028] Lamkemeyer P Laxa M Collin V 2006 Peroxiredoxin Q of *Arabidopsis thaliana* is attached to the thylakoids and functions in context of photosynthesis. The Plant Journal 45, 968–981 16507087 10.1111/j.1365-313X.2006.02665.x

[CIT0029] Lemaire SD Collin V Keryer E Quesada A Miginiac-Maslow M 2003 Characterization of thioredoxin *y*, a new type of thioredoxin identified in the genome of *Chlamydomonas reinhardtii* . FEBS Letters 543, 87–92 12753911 10.1016/s0014-5793(03)00416-2

[CIT0030] Lemaire SD Quesada A Merchan F Corral JM Igeno MI Keryer E Issakidis-Bourguet E Hirasawa M Knaff DB Miginiac-Maslow M 2005 NADP-malate dehydrogenase from unicellular green alga *Chlamydomonas reinhardtii*. A first step toward redox regulation? Plant Physiology 137, 514–521 15579663 10.1104/pp.104.052670 PMC1065352

[CIT0031] Li Q Xie QG Smith-Becker J Navarre DA Kaloshian I 2006 *Mi-1*-mediated aphid resistance involves salicylic acid and mitogen-activated protein kinase signaling cascades. Molecular Plant-Microbe Interactions 19, 655–664 16776299 10.1094/MPMI-19-0655

[CIT0032] Lilley RM Walker DA 1974 An improved spectrophotometric assay for ribulose-bisphosphate carboxylase. Biochimica et Biophysica Acta 358, 226–229 4368401 10.1016/0005-2744(74)90274-5

[CIT0033] Liu Y Schiff M Dinesh-Kumar SP 2002 *a* Virus-induced gene silencing in tomato. The Plant Journal 31, 777–786 12220268 10.1046/j.1365-313x.2002.01394.x

[CIT0034] Liu Y Schiff M Marathe R Dinesh-Kumar SP 2002 *b* Tobacco *Rar1*, *EDS1* and *NPR1*/*NIM1* like genes are required for *N*-mediated resistance to tobacco mosaic virus. The Plant Journal 30, 415–429 12028572 10.1046/j.1365-313x.2002.01297.x

[CIT0035] Livak KJ Schmittgen TD 2001 Analysis of relative gene expression data using real-time quantitative PCR and the 2^–ΔΔCT^ method. Methods 25, 402–408 11846609 10.1006/meth.2001.1262

[CIT0036] Long SP Zhu X Naidu SL Ort DR 2006 Can improvement in photosynthesis increase crop yields? Plant, Cell and Environment 29, 315–330 10.1111/j.1365-3040.2005.01493.x 17080588

[CIT0037] Luo T Fan TT Liu YN Rothbart M Yu J Zhou SX Grimm B Luo MZ 2012 Thioredoxin redox regulates ATPase activity of magnesium chelatase CHLI subunit and modulates redox-mediated signaling in tetrapyrrole biosynthesis and homeostasis of reactive oxygen species in pea plants. Plant Physiology 159, 118–130 22452855 10.1104/pp.112.195446 PMC3375955

[CIT0038] Marchand C Le Marechal P Meyer Y Decottignies P 2006 Comparative proteomic approaches for the isolation of proteins interacting with thioredoxin. Proteomics 6, 6528–6537 17163439 10.1002/pmic.200600443

[CIT0039] Marcus F Chamberlain SH Chu C Masiarz FR Shin S Yee BC Buchanan BB 1991 Plant thioredoxin *h*: an animal-like thioredoxin occurring in multiple cell compartments. Archives of Biochemistry and Biophysics 287, 195–198 1897989 10.1016/0003-9861(91)90406-9

[CIT0040] Marty L Siala W Schwarzländer M Fricker MD Wirtz M Sweetlove LJ Meyer Y Meyer AJ Reichheld JP Hell R 2009 The NADPH-dependent thioredoxin system constitutes a functional backup for cytosolic glutathione reductase in *Arabidopsis* . Proceedings of the National Academy of Sciences, USA 106, 9109–9114 10.1073/pnas.0900206106 PMC2690020 19451637

[CIT0041] Meyer AJ Brach T Marty L Kreye S Rouhier N Jacquot JP Hell R 2007 Redox-sensitive GFP in *Arabidopsis thaliana* is a quantitative biosensor for the redox potential of the cellular glutathione redox buffer. The Plant Journal 52, 973–986 17892447 10.1111/j.1365-313X.2007.03280.x

[CIT0042] Meyer Y Reichheld JP Vignols F 2005 Thioredoxins in *Arabidopsis* and other plants. Photosynthesis Research 86, 419–433 16307307 10.1007/s11120-005-5220-y

[CIT0043] Meyer Y Siala W Bashandy T Riondet C Vignols F Reichheld JP 2008 Glutaredoxins and thioredoxins in plants. Biochimica et Biophysica Acta 1783, 589–600 18047840 10.1016/j.bbamcr.2007.10.017

[CIT0044] Montrichard F Alkhalfioui F Yano H Vensel WH Hurkman WJ Buchanan BB 2009 Thioredoxin targets in plants: the first 30 years. Journal of Proteomics 72, 452–474 19135183 10.1016/j.jprot.2008.12.002

[CIT0045] Motohashi K Kondoh A Stumpp MT Hisabori T 2001 Comprehensive survey of proteins targeted by chloroplast thioredoxin. Proceedings of the National Academy of Sciences, USA 98, 11224–11229 10.1073/pnas.191282098 PMC58711 11553771

[CIT0046] Müssig C 2005 Brassinosteroid-promoted growth. Plant Biology 7, 110–117 15822006 10.1055/s-2005-837493

[CIT0047] Müssig C Fischer S Altmann T 2002 Brassinosteroid-regulated gene expression. Plant Physiology 129, 1241–1251 12114578 10.1104/pp.011003 PMC166518

[CIT0048] Muthuramalingam M Dietz K-J Ströher E 2010 Thiol–disulfide redox proteomics in plant research. Methods in Molecular Biology 639, 219–238 20387049 10.1007/978-1-60761-702-0_13

[CIT0049] Nakano Y Asada K 1981 Hydrogen peroxide is scavenged by ascorbate specific peroxidase in spinach chloroplasts. Plant and Cell Physiology 22, 867–880

[CIT0050] Navrot N Collin V Gualberto J Gelhaye E Hirasawa M Rey P Knaff DB Issakidis E Jacquot JP Rouhier N 2006 Plant glutathione peroxidases are functional peroxiredoxins distributed in several subcellular compartments and regulated during biotic and abiotic stresses. Plant Physiology 142, 1364–1379 17071643 10.1104/pp.106.089458 PMC1676047

[CIT0051] Nishizawa AN Buchanan BB 1981 Enzyme regulation in C_4_ photosynthesis. Purification and properties of thioredoxin-linked fructose bisphosphatase and sedoheptulose bisphosphatase from corn leaves. Journal of Biological Chemistry 256, 6119–6126 6263905

[CIT0052] Rao MV Ormrod DP 1995 Ozone exposure decreases UVB sensitivity in a UVB-sensitive flavonoid mutant of *Arabidopsis* . Photochemistry and Photobiology 61, 71–78 7899496 10.1111/j.1751-1097.1995.tb09245.x

[CIT0053] Rouhier N Gelhaye E Gualberto JM 2004 Poplar peroxiredoxin Q. A thioredoxin-linked chloroplast antioxidant functional in pathogen defense. Plant Physiology 134, 1027–1038 14976238 10.1104/pp.103.035865 PMC389925

[CIT0054] Sasaki Y Kozaki A Hatano M 1997 Link between light and fatty acid synthesis: thioredoxin-linked reductive activation of plastidic acetyl-CoA carboxylase. Proceedings of the National Academy of Sciences, USA 94, 11096–11101 10.1073/pnas.94.20.11096 PMC23628 9380765

[CIT0055] Scheibe R Fickenscher K Ashton AR 1986 Studies on the mechanism of the reductive activation of NADP-malate dehydrogenase by thioredoxin *m* and low molecular weight thiols. Biochimica et Biophysica Acta 870, 191–197

[CIT0056] Schürmann P Buchanan BB 2008 The ferredoxin/thioredoxin system of oxygenic photosynthesis. Antioxidants and Redox Signaling 10, 1235–1273 18377232 10.1089/ars.2007.1931

[CIT0057] Schwarz O Schurmann P Strotmann H 1997 Kinetics and thioredoxin specificity of thiol modulation of the chloroplast H^+^-ATPase. Journal of Biological Chemistry 272, 16924–16927 9202002 10.1074/jbc.272.27.16924

[CIT0058] Ströher E Dietz KJ 2008 The dynamic thiol–disulphide redox proteome of the *Arabidopsis thaliana* chloroplast as revealed by differential electrophoretic mobility. Physiologia Plantarum 133, 566–583 18433418 10.1111/j.1399-3054.2008.01103.x

[CIT0059] Thormählen I Ruber J Roepenack-Lahaye EV Ehrlich S-M Massot V Hümmer C Tezycka J Issakidis-Bourguet E Geigenberger P 2013 Inactivation of thioredoxin *f*1 leads to decreased light activation of ADP-glucose pyrophosphorylase and altered diurnal starch turnover in leaves of *Arabidopsis* plants. Plant, Cell and Environment p36, 16–29 10.1111/j.1365-3040.2012.02549.x 22646759

[CIT0060] Vieira Dos Santos C Laugier E Tarrago L Massot V Issakidis–Bourguet E Rouhier N Rey P 2007 Specificity of thioredoxins and glutaredoxins as electron donors to two distinct classes of *Arabidopsis* plastidial methionine sulfoxide reductases B. FEBS Letters 581, 4371–4376 17761174 10.1016/j.febslet.2007.07.081

[CIT0061] von Caemmerer S Farquhar GD 1981 Some relationships between the biochemistry of photosynthesis and the gas exchange of leaves. Planta 153, 376–387 24276943 10.1007/BF00384257

[CIT0062] Wang H Wang SB Lu YQ Alvarez S Hicks LM Ge XC Xia YJ 2012 Proteomic analysis of early-responsive redox-sensitive proteins in *Arabidopsis* . Journal of Proteome Research 11, 412–424 22050424 10.1021/pr200918f PMC3253204

[CIT0063] Wenderoth I Scheibe R von Schaewen A 1997 Identification of the cysteine residues involved in redox modification of plant plastidic glucose-6-phosphate dehydrogenase. Journal of Biological Chemistry 272, 26985–26990 9341136 10.1074/jbc.272.43.26985

[CIT0064] Wolosiuk RA Ballicora MA Hagelin K 1993 The reductive pentose phosphate cycle for photosynthetic CO_2_ assimilation: enzyme modulation. FASEB Journal 7, 622–637 8500687 10.1096/fasebj.7.8.8500687

[CIT0065] Wong JH Cai N Balmer Y Tanaka CK Vensel WH Hurkman WJ Buchanan BB 2004 Thioredoxin targets of developing wheat seeds identified by complementary proteomic approaches. Phytochemistry 65, 1629–1640 15276458 10.1016/j.phytochem.2004.05.010

[CIT0066] Wu CY Trieu A Radhakrishnan P 2008 Brassinosteroids regulate grain filling in rice. The Plant Cell 20, 2130–2145 18708477 10.1105/tpc.107.055087 PMC2553602

[CIT0067] Xia XJ Huang LF Zhou YH Mao WH Shi K Wu JX Asami T Chen ZX Yu JQ 2009 *a* Brassinosteroids promote photosynthesis and growth by enhancing activation of Rubisco and expression of photosynthetic genes in *Cucumis sativus.* Planta 230, 1185–1196 19760261 10.1007/s00425-009-1016-1

[CIT0068] Xia XJ Wang YJ Zhou YH Tao Y Mao WH Shi K Asami T Chen ZX Yu JQ 2009 *b* Reactive oxygen species are involved in brassinosteroid-induced stress tolerance in cucumber. Plant Physiology 150, 801–814 19386805 10.1104/pp.109.138230 PMC2689980

[CIT0069] Yu JQ Huang LF Hu WH Zhou YH Mao WH Ye SF Nogues S 2004 A role for brassinosteroids in the regulation of photosynthesis in *Cucumis sativus* . Journal of Experimental Botany 55, 1135–1143 15107450 10.1093/jxb/erh124

[CIT0070] Zhang N Portis AR Jr 1999 Mechanism of light regulation of Rubisco: a specific role for the larger Rubisco activase isoform involving reductive activation by thioredoxin-f. Proceedings of the National Academy of Sciences, USA 96, 9438–9443 10.1073/pnas.96.16.9438 PMC17801 10430961

