# Supplementary material for: Chloroplastic thioredoxin-f and thioredoxin-m1/4 play important roles in brassinosteroids-induced changes in CO2 assimilation and cellular redox homeostasis in tomato
Source: J Exp Bot. 2014 May 20;65(15):4335–47. doi: 10.1093/jxb/eru207 (PMC4112637; doi:10.1093/jxb/eru207)
Supplement: Supplementary Data [file supp_65_15_4335__index.html]

Chloroplastic thioredoxin-f and thioredoxin-m1/4 play important roles in brassinosteroids-induced changes in CO2 assimilation and cellular redox homeostasis in tomato — Chloroplastic thioredoxin-f and thioredoxin-m1/4 play important roles in brassinosteroids-induced changes in CO2 assimilation and cellular redox homeostasis in tomato — Supplementary Data 

# Chloroplastic thioredoxin-*f* and thioredoxin-*m*1/4 play important roles in brassinosteroids-induced changes in CO2 assimilation and cellular redox homeostasis in tomato

## Supplementary Data

Data files

**Files in this Data Supplement:**

- Supplementary Data - Supplementary Data
