# Supplementary material for: Chloroplastic thioredoxin-f and thioredoxin-m1/4 play important roles in brassinosteroids-induced changes in CO2 assimilation and cellular redox homeostasis in tomato
Source: J Exp Bot. 2014 May 20;65(15):4335–47. doi: 10.1093/jxb/eru207 (PMC4112637; doi:10.1093/jxb/eru207)

Supplemental Table S1. PCR primers designed for vector construction.

Vector	Forward primer	Reverse primer
pTRV2-*TRX-f*	5’-CGGCGCTCGAG ATATTTCTTCTTTTTTTT- 3’	5’-GGCGCGAGCTC AAGAATATGAGAATTTAGCT- 3’
pTRV2-*TRX-m2*	5’-CGGCGCTCGAG TACAATGGTGCGCGCATA- 3’	5’-GGCGCGAGCTC TAGTAGAATATTACTTCA- 3’
pTRV2-*TRX-m1/4*	5’-CGGCGCTCGAG CCTGGTGTTAATGATCAAT- 3’	5’-GGCGCGAGCTC TTACAAGAATTTTTCTAT- 3’
pTRV2-*TRX-x*	5’-CGGCGCTCGAG TATTCAGGGAAAAGTTCA- 3’	5’-GGCGCGAGCTC TGCATAAATACGATCATC- 3’
pTRV2-*TRX-y*	5’-CGGCGCTCGAG TCTTAGCGTGCTGCCTTT- 3’	5’-GGCGCGAGCTC TACATTGAACATCAGTTG- 3’

Supplemental Table S2. Gene-specific primers designed for quantitative real-time RT-PCR.

Gene	Accession numbers	Forward primer	Reverse primer
actin	Sl11g005330	5’-TGTCCCTATTTACGAGGGTTATGC- 3’	5’-CAGTTAAATCACGACCAGCAAGAT- 3’
RCA	Sl10g086580	5’-CTGTTGGTCATCCGATGTGT- 3’	5’-CCCAAGGTTTCAAACAGGAA- 3’
*rbc*L	Sl01g007330	5’-ACCGCAAATACTACCTTGGC- 3’	5’-CCACCAGACATACGTAACGC- 3’
*rbc*S	Sl02g063150	5’-TTGCTTGGAATTCGAGACTG- 3’	5’-CTCTTGAACCTCAGCCAACA- 3’
FBPase	Sl02g062340	5’-GAAGAGAAATGGCATCAGCA- 3’	5’-AGTGAGTCCAGAAGGATGGG- 3’
SBPase	Sl05g052600	5’-AGTTGGTGCTGCTGTTTGAG- 3’	5’-TTCGCGATGCTCTAGAAAGA- 3’
PGK	Sl07g066610	5’-GAAGGGCAAGAAAGTCTTCG- 3’	5’-AGTGTTTGATGGTAGGGATGG- 3’
PRK	Sl08g076220	5’-GGGATATGGCTGAAAGAGGA- 3’	5’-CTGAGTTGGGAGCACTTCAA- 3’
GAPDH	Sl03g111010	5’-GATTGGAGAGGTGGAAGAGC- 3’	5’-ACCACGGACACATCAACAGT- 3’
TRX-f	Sl05g056300	5’-CACAGTGGTGTGGTCCTTGT- 3’	5’-TTTGCTAGTGGCCTGTTGTC- 3’
TRX-m2	Sl10g006970	5’-GTCGATCCTCGGCAGTTAAT- 3’	5’-TTTCCTGAGCTTCACAGACG- 3’
TRX-m1/4	Sl12g013810	5’-CCGTCGTTGTCTCCTGTTT- 3’	5’-GCCTCACAGACGATTCTTCC- 3’
TRX-x	Sl01g008250	5’-CCGTTGCTGAGGAGTACAAA- 3’	5’-TCTGGGACTTCCTTTCCATC- 3’
TRX-y	Sl04g071560	5’-CGTCTTGTTCCTCTGGTTGA- 3’	5’-TAGGAACCATGAACTGGCAA- 3’

Supplemental Figure S1. Relative mRNA abundance of *TRX-f*, *TRX-m2*, *TRX-m1/4*, *TRX-x* and *TRX-y* in respective virus-induced gene silencing (VIGS) plants. Data are the means of the 5th leaf of six silenced plants (±SD). Relative gene expression for each *TRX* gene was calculated as the pTRV plants (control)=1.

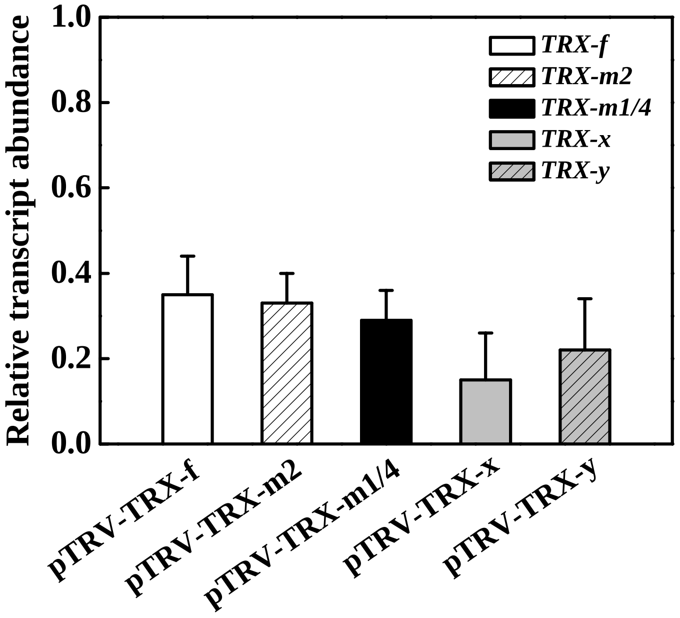

Supplemental Figure S2. Changes in maximum quantum yield of PSII (*F*v/*F*m) in the leaves of pTRV and various partially *TRX*-silenced plants. *F*v/*F*m was measured with imaging PAM (IMAG-MAXI; Heinz Walz, Effeltrich, Germany) after whole plants were dark adapted for 30 min. Minimal fluorescence (*F*o) was measured during the weak measuring pulses and maximal fluorescence (*F*m) was measured by a 0.8-s pulse light at about 4,000 μmol m^{-2} s^{-1}. (*F*m-*F*o)/*F*m = *F*v/*F*m. Data are the means of four replicates with SDs. Means followed by the same letter are not significantly different according to Tukey's test ($P < 0.05$).

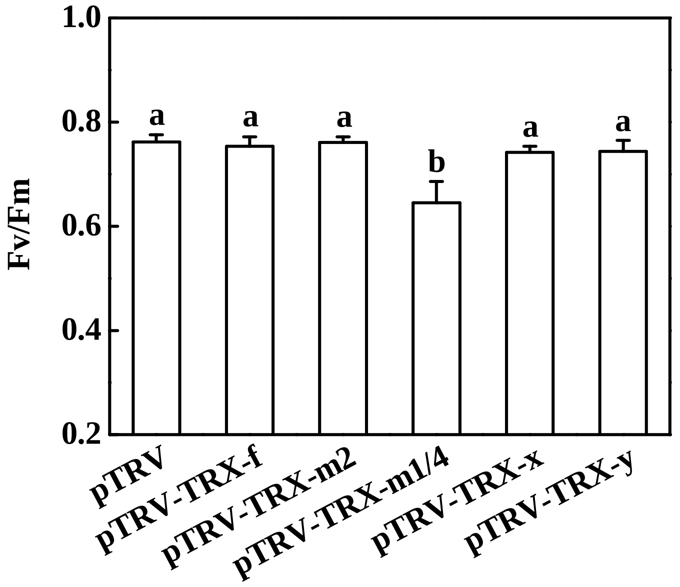

Supplement: Supplementary Data [file supp_eru207_jexbot120048_file001.pdf]
